# Gender Differences in Contraceptive Self-Efficacy: A Cross-Sectional Study of South Korean College Students

**DOI:** 10.3390/ijerph17093142

**Published:** 2020-04-30

**Authors:** Eun-Young Jun, Hyunjin Oh

**Affiliations:** 1 Department of Nursing, Daejeon University, Daejeon 34520, Korea; 1991young1@hanmail.net; 2 College of Nursing, Gachon University, Incheon 21936, Korea

**Keywords:** college students, contraception, self-efficacy, sex differences

## Abstract

Purpose: Healthy sexuality is an important issue in the transition to adulthood. To maintain healthy sexuality, contraceptive self-efficacy could be the most significant predictor of safe sexuality. The purpose of this study was to explore the relationship between gender roles and the healthy sexuality of South Korean college students. Method: A cross-sectional study through self-report questionnaires was administered to college students in South Korea. In total, 499 students completed the demographic information questionnaire, the Sexual Attitudes Scale, Sexual Autonomy Scale, and Condom Self-Efficacy Scale. Results: A multiple regression analysis indicated that the contraceptive self-efficacy of male students was predicted by their junior year and sexual autonomy, while that of the females was predicted by their senior year and sexual autonomy. Conclusion: Sexual education focusing on sexual autonomy should be provided for the safe and healthy sexual expression of college students.

## 1. Introduction

Healthy sexuality involves sexual wellness―a concept subjectively and individually defined. The World Health Organization notes that “sexual health requires a positive approach to sexuality and sexual relationships, as well as the possibility of having pleasurable and safe sexual experiences, free of coercion, discrimination, and violence” [1]. The perception of healthy or harmful sexual experiences should be examined from the perspective of different genders and the cultural context where individuals live [2].

In order to maintain a healthy practice of sexuality, contraceptive self-efficacy could be the most significant predictor of contraceptive use of sexually active college students. It is also important to elucidate the underlying gender differences in the sexual attitudes, autonomy, and contraceptive self-efficacy of young adults who are transitioning to adulthood. Contraceptive self-efficacy refers to the belief that a person can control sexual and contraceptive situations to prevent unwanted pregnancy and sexually transmitted disease [3]. Contraceptive self-efficacy was developed to measure the theory of self-efficacy in sexual health, focusing on adolescents who have difficulty in saying no [4]. Since an individual’s behavior is generally determined by one’s perceived self-efficacy, existing literature has established evidence about the relationships between contraceptive self-efficacy and contraceptive behavior [5]. In this context, healthy sexuality implies positive attitudes, high sexual autonomy, and contraceptive self-efficacy.

Attitudes are consistent sets of thoughts, emotions, and behaviors about an object that are constructed from one’s own experiences, educational background, relationships, and cultural background, while sexual attitudes imply positive or less positive perspectives and tendencies when cognitively approaching sexuality [6]. A meta-analysis review showed that there are gender differences in sexual attitudes as men have more explicit and permissive attitudes toward sexuality that are closely related to active sexual desire [7,8]. Under the traditional Confucian ideology about gender norms, women in South Korea have a stereotype of passiveness and obedience toward men and sexuality [9]. Although society is changing and the practice of pre-marital sex is common in the South Korean society, it is still difficult for young and unmarried women to actively reveal their sexual desire and explicit attitudes [10].

Sexual autonomy, as one of the fundamental human rights, is an expression of one’s feelings and will concerning all sexual experiences [11]. Sexual autonomy is an essential concept learned in the transition to sexual maturity by young adults [12]. At this time, individual sexual autonomy needs to be reconstructed and redefined. It implies both the freedom to give oneself to another and the ability to own or control one’s own body. In other words, as a conscious and willing choice for sexual health, adults enjoy sexual autonomy in addition to avoiding sexual problems and maintaining self-esteem as sexual beings [12,13]. It seems that adults may have a double standard depending on social standards or norms. In some cultures with a conservative stance on sexuality, few young adults are assertive in sexual situations; there is a sexual double standard where men should lead sex-related decisions, and their sexual practices are widely accepted [9]. A person with higher sexual autonomy can control their sexual desires, urges, and situations for their sexual health [14].

Diverse socio-demographic factors, such as culture, age, economic status, employment, and education, have been studied in relation to sexual health [9,10,14,15,16], but a comprehensive approach in understanding the gender differences of young adults in South Korea is still lacking. Research shows that gender plays an important role in configuring the sexual health beliefs, attitudes, and behaviors of adolescents [15,17]. College students are transitioning from adolescence to adulthood. In South Korean, by the age of 19, a person is considered a legal adult. There is limited sexual health discourse and research about gender as a key demographic characteristic that affects the difference between men and women in the transitional phase. Sexual attitudes, autonomy, and contraceptive self-efficacy are the key social constructs, and fundamental determinants of young adults’ sexual health since societal and cultural stereotypes and norms result in gender-specific expectations and patterns [16,18,19].

The purposes of the studies were:

1. To explore the sexual attitudes, sexual autonomy, and contraceptive self-efficacy of South Korean college students;
2. To identify the factors that influence the contraceptive self-efficacy of college students by gender.

## 2. Methods

A descriptive cross-sectional study was conducted to examine the factors influencing the contraceptive self-efficacy of South Korean college students. This study attempted to provide basic data for the development of well-grounded sexual health education programs for college students.

### 2.1. Setting and Sample

This study was conducted at Daejeon University, with 10,000 students in South Korea. A convenience sample of college students was recruited from the school by posting announcements during school events, and those who voluntarily visited the booth participated in the study. They were informed about the purpose of the research and were assured that their participation would be voluntary and anonymous. A power analysis was conducted a priori using G * power software version 3.1.7, that indicated that at least 118 subjects would be required to detect a medium effect size (f^2^) of 0.15, with 80% of power, a two-sided α of 0.05, and 10 potential predictors. A total of 512 students were eligible. Of these, 13 students that had missing data were excluded. Thus, a final sample of 499 participants were included in the analyses. 

### 2.2. Measurements

#### 2.2.1. Demographic Characteristics

Self-reported demographic characteristics were collected; these included their year level, family’s economic status, parental status, current residence, dating experience, number of dating experiences, the average length of dating, sources of information on sexuality, and level of sexual contact.

#### 2.2.2. Sexual Attitude

The sexual attitude was measured using the Sexual Attitudes Scale developed by Hendrick and Hendrick [8] for young adults and was translated and modified by Choi [20]. The Sexual Attitudes Scale is a multi-dimensional instrument with 43 items that have sub-concepts of permissiveness (casual sexuality), sexual practice (responsible, tolerant sexuality), communion (idealistic sexuality), and instrumentality (biological, utilitarian sexuality). Choi [20) tested the validity of the sexual attitude scale by administering it to high school students and selected 25 items. In this study, 23 items were used, except for two that did not fit the South Korean situation. Each item is rated on a five-point Likert scale (1 = strongly disagree, 5 = strongly agree), and the range of possible scores for this scale is 23–115, with a higher score indicating more openness in the person’s sexual attitude. At the time of development and modification, the Cronbach’s alpha was 0.75–0.94 [7,8], 64–85 [20]. This study had a Cronbach’s alpha of 0.89.

#### 2.2.3. Sexual Autonomy

The Sexual Autonomy Scale was developed for college students but was validated as an effective tool for both college students and unmarried men and women in South Korea [12]. This tool has sub-concepts of control and coping, with 13 questions measured on a five-point Likert scale (1 = strongly disagree, 5 = strongly agree). The range of possible scores for this scale is 13–65, with higher scores indicating a higher degree of sexual autonomy. Cronbach’s alpha in the original study was 0.86 [12], and 0.90 in this study.

#### 2.2.4. Contraceptive Self-Efficacy

Contraceptive self-efficacy was measured using the Condom Self-Efficacy Scale developed [21] and modified by Hwang and Chung to fit the situation of contraception [22]. This scale consists of 12 items, and each item is rated on a five-point Likert scale (1 = strongly disagree, 5 = strongly agree). The range of possible scores for this scale is 12–60, and a higher score indicates higher contraceptive self-efficacy. At the time of development and modification, Cronbach’s alpha was 0.70 [21] and 0.76 [22], respectively. This study had a Cronbach’s alpha of 0.80.

### 2.3. Data Collection and Ethical Consideration

The data were collected using self-report questionnaires from May 9 to 13, 2016. Questionnaires were distributed when participants visited the booth during school events. Research assistants explained the purpose of the research, the procedures, and the voluntary nature of the study to potential participants. In order to foster participation and reduce discomfort, research assistants distributed and helped participants fill out the questionnaires, which took approximately 15 min each to complete. 

This study was approved by the Institutional Review Board of Daejeon University (1040647-201602-HR-002-03); all the participants signed written informed consent forms before recruitment. The participants were assured of confidentiality and anonymity prior to the beginning of the study and were reminded of their right to refuse to participate or withdraw from the investigation.

### 2.4. Statistical Analysis

Statistical analyses were performed using IBM SPSS 22.0. Demographic characteristics, sexual attitude, sexual autonomy, and contraceptive self-efficacy were presented based on the frequency, mean, and standard deviation. The differences in sexual attitude, sexual autonomy, and contraceptive self-efficacy based on demographic characteristics were evaluated using *t*-tests and analyses of variance with Scheffé post hoc test. The association between contraceptive self-efficacy, sexual attitude, and autonomy was examined using Pearson’s correlation. Factors influencing contraceptive self-efficacy were analyzed using hierarchical multiple regression.

## 3. Results

### 3.1. Demographics, Sexual Attitude, Sexual Autonomy, and Contraceptive Self-Efficacy

Participants comprised 253 men (50.3%) and 246 women (49.7%); according to the academic year, most of them were freshmen while seniors were the fewest. Most belonged to a middle-class family (83.8%) and still had parents (91.4%), but only about one third lived in their parents’ home.

In total, 403 participants (80.8%) had experience dating, a mean dating experience of 2.91 times, an average dating length of less than 12 months (84.0%). Among the participants, 42.6% had experienced sexual intercourse, and there were no differences in gender. Their main source of sexuality information was media (36.6%). The mean score on the sexual attitude and autonomy scales was 67.80 (SD = 12.65) and 51.41 (SD = 7.63), respectively, and 45.94 (SD = 6.64) on the Contraceptive Self-efficacy scale (Table 1).

### 3.2. Differences in Sexual Attitude, Sexual Autonomy, and Contraceptive Self-Efficacy

Sexual attitude scores were significantly associated with gender (F = 6.34, *p* < 0.001) and source of sexuality information (F = 7.24, *p* < 0.001). Sexual autonomy scores were significantly associated with gender (F = − 3.62, *p* < 0.001), year level (F = 5.38, *p* = 0.001), and average dating experience (F = 3.92, *p* = 0.009). Contraceptive self-efficacy scores were significantly associated with gender (F = −4.60, *p* < 0.001), year level (F = 7.19, *p* < 0.001), and dating experience (F = −2.52, *p* = 0.012) (Table 2).

The relationships between contraceptive self-efficacy, sexual attitude, and sexual autonomy were analyzed using Pearson’s correlation. Contraceptive self-efficacy was found to correlate significantly and positively with sexual attitude (r = 0.09, *p* = 0.041) and sexual autonomy (r = 0.61, *p* < 0.001).

### 3.3. Factors Influencing Contraceptive Self-Efficacy 

Multiple regression analysis was conducted to examine the factors influencing contraceptive self-efficacy. The residual analysis confirmed that the assumptions of multicollinearity, linearity, normality, and equality of variance were met in the regression equation. To identify the factors influencing contraceptive self-efficacy, because a gender difference was shown, hierarchical regression analysis was performed by gender. In this study, independent factors were assessed for any significant impact on contraceptive self-efficacy via univariate analysis. To develop the contraceptive self-efficacy model, we entered demographic characteristics (year level, dating experience, and sources of sexuality information) in the first block and sexual attitude in the second block. Finally, we entered sexual autonomy in the third block to examine the effects of the contraceptive self-efficacy. 

In males, the addition of sexual autonomy to the second block equation resulted in 45.5% of explained variance in contraceptive self-efficacy, showing that sexual autonomy was a significant factor influencing contraceptive self-efficacy (β = 0.63, *p* < 0.001), and the model was adequate (F = 36.03, *p* < 0.001). In females, the addition of sexual autonomy to the second block equation resulted in 30.8% of the explained variance in contraceptive self-efficacy, which was the same as that in males, indicating that sexual autonomy was a significant factor influencing contraceptive self-efficacy (β = 0.52, *p* < 0.001) and that the model was adequate (F = 19.15, *p* < 0.001) (Table 3).

## 4. Discussion

In this study, significant variations in sexual attitudes, sexual autonomy, and contraceptive self-efficacy by demographic characteristics emerged. Female students tended to have higher sexual autonomy and contraceptive self-efficacy while male students have higher sexual attitudes, meaning they are more active and have an open tendency toward sexuality. These results are consistent with previous studies that sexual autonomy [23] and contraceptive self-efficacy [22] are higher in women than men, and sexual attitude is higher in men than women [24].

In this study, second-year college students showed a relatively high increased level of sexual autonomy and contraceptive self-efficacy. In South Korea, second-year students have the opportunity to experience a variety of sexual experiences since it is generally considered the legal age of adulthood. They may be exposed to more sexual experiences than freshmen, along with frequent opportunities to consider sexual autonomy and contraception, thus, resulting in a high increase in sexual autonomy and contraceptive self-efficacy in the second year. Contraceptive self-efficacy was high in males in the second year and females in the fourth year. In South Korea, most male students go to the military during their sophomore year, and situational factors such as enlistment may have affected this. Education and intervention for healthy sexuality may be provided during this period.

Students with dating experience tended to have a high level of sexual autonomy and contraceptive self-efficacy. Sexual autonomy and contraceptive self-efficacy were highest when the students got sexuality information from their teachers and parents, while sexual attitude was highest when students considered friends as their main information resources. Although there is no statistically significant difference between existing sexual education through school or parents, sexual autonomy can be seen as an effect of education from older generations such as parents and teachers. These differences may help explain behavioral and educational perspectives of healthy sexuality of the young adult population in South Korea. The continuing general sexual education program in schools had an effect on sexual knowledge but not on sexual attitude [25]. Therefore, given that characteristics of sexual attitudes related to psychological and relational aspects are more influenced by friends and the media, it can be seen that traditional sexual education is not enough to change existing attitudes regarding healthy sexuality.

Consistent with prior work by Geer and Robertson [26], which found that women had less positive and implicit attitudes toward sexuality than men did, we found that male students had positive and open attitudes toward sexuality. Interestingly, we found that female students were relatively more likely to have higher sexual autonomy and contraceptive self-efficacy. This implies that women may still have fewer positive attitudes than males regarding sexual activities, as they seem to be subjected more to cultivating responsible attitudes for sexuality, compared to men. A previous study presented that girls may not feel comfortable with sexual decision making within sexual relationships or safe sexual intercourse, and they feel pressured to take a passive role because of the norms of proper femininity [15,27]. This may cause difficulties as women are at risk for pregnancy in unsafe or high-risk sexual behaviors. Female college students have higher sexual autonomy and contraceptive self-efficacy than males, implying that they were more responsible for their sexual behavior. Interestingly the level of sexual attitudes, sexual autonomy, and contraceptive self-efficacy greatly increased in the second year of college students. In South Korea, most adolescents are under intense competition and pressure in academic performance [28]. Becoming a college student means they have enough power and control to do what they want to do [10]. The sudden increase in attitude, autonomy, and contraceptive self-efficacy toward sexuality means that there is room to educate and perform active intervention when they enter college. Under the premise that sexual education has been continuously conducted since infancy, it focuses on sexual harassment and sexual violence that reflects the gender-related issues of older generations [10]. Therefore, given the significant increase in the level of sexual autonomy and self-efficacy for contraception in the second year of college, education is required to embed this into their broader sexual health [29,30].

In this study, when sexual autonomy was added to the analysis of both men and women, the rationale behind contraceptive self-efficacy increased significantly. Therefore, sexual autonomy was identified as a major factor influencing contraceptive self-efficacy for both genders. Sexual autonomy expresses a willingness to take care of one’s body, and it gives individuals sexual pride or identity. It is the willingness to sexual relationship and responsibility toward one’s body and the ability to act on oneself [12,13]. Contraceptive self-efficacy is closely related to sexual autonomy and seems to be related to conceptual and attribute similarity. Healthy sexuality requires the management of sexual health that considers physical, psychological, and relational aspects. In this sense, sexual autonomy is an important concept that can reflect the characteristics of healthy sexuality. Therefore, sexual education focusing on sexual autonomy should be provided for the safe and healthy sexuality of college students. To this end, education should not only be an attribute of sexual autonomy but also support for the establishment of self-identity, a task of college students. In other words, the sexual education of college students should be perception and value-centered, and because the owners of their sexual health are themselves, they should experience sexual education that focuses on empowering them to manage their own sexual health.

This study shows that educational interventions promoting sexual autonomy must be implemented in order to increase contraceptive self-efficacy and to have safe and healthy sexuality. Based on the results of this study, college students need education on healthy sexuality enabling them to recognize their value as a sexual being and to control their sexual behavior.

This study provides basic data for the development of sexual health education programs for college students, but there are several limitations that must be taken into consideration when drawing conclusions. Even though the sample size was large enough to run a statistical analysis, the participants in this study were recruited from only one university. Generalizability may be limited to similar characteristics of the study population and may not extend to all college students. Most of the participants in this study were those who visited the booth during school events and, thus, may be more likely to be interested in sexuality than students who did not visit. Although it was not intended, there was little participation in the senior, and they seemed to be less interested in school events or due to preparation activities and classes for employment. It is possible that selection bias in sampling occurred and affected study findings. Therefore, further studies should be considered using stratified sampling by grade level and gender so that all students from freshman to senior can participate uniformly.

## 5. Conclusions

In this descriptive study, we identified the factors influencing contraceptive self-efficacy of South Korean college students. The findings of this study demonstrated that college students had differences in sexual autonomy and contraceptive self-efficacy by year level, especially second-year college students with an increased level of sexual autonomy and contraceptive self-efficacy. Furthermore, they had differences in sexual attitude, sexual autonomy, and contraceptive self-efficacy by gender, and sexual autonomy was the most important variable in contraceptive self-efficacy for both genders.

Sexual health education is necessary for first-year college students who are socially recognized as adults and are exposed to increasing opportunities for sexual experience. Therefore, it should provide not only the right values for sexuality but also sexual health-related issues, such as increasing contraceptive self-efficacy, and consider gender differences. In addition, it is suggested that active education be provided so that they may consult with experts regarding their healthy sexuality. Further studies from diverse schools or areas might be helpful if differences emerge, and the use of qualitative methodologies is recommended to investigate in-depth a college student’s experiences related to sexual health.

## Figures and Tables

**Table 1:** Participant characteristics (n = 499).

Characteristics	Categories	Totaln (%)	Male (n = 253) n (%)	Female (n = 246) n (%)	M ± SD	Ideal Range (min~max)
Grade	Freshman	188 (37.7)	102 (40.3)	86 (34.9)		
	Sophomore	131 (26.3)	60 (23.7)	71 (28.9)		
	Junior	119 (23.8)	61 (24.1)	58 (23.6)		
	Senior	61 (12.2)	30 (11.9)	31 (12.6)		
Family economic status	High	39 (7.8)	24 ( 9.5)	15 (6.1)		
	Middle	418 (83.8)	202 (79.8)	216 (87.8)		
	Low	42 (8.4)	27 (10.7)	15 (6.1)		
Parental status	Having parents	456 (91.4)	232 (91.7)	224 (91.1)		
	Parent or not having parents	43 (8.6)	21 (8.3)	22 (8.9)		
Current residence	Parents’ home	179 (35.9)	79 (31.2)	100 (40.7)		
	Campus residence hall	92 (18.4)	48 (19.0)	44 (17.9)		
	Off-campus housing	228 (45.7)	126 (49.8)	102 (41.5)		
Date experience	No	96 (19.2)	49 (19.4)	47 (19.1)		
	Yes	403 (80.8)	204 (80.6)	199 (80.9)		
Number of dating experience †	1	89 (22.1)	46 (22.5)	43 (21.6)	2.91 ± 3.53	
	2~3	170 (42.2)	74 (36.3)	96 (48.2)		
	4~5	91 (22.6)	51 (25.0)	40 (20.1)		
	≥6	53 (13.1)	33 (16.2)	20 (10.1)		
Average dating length (month) ^†^	≤3	125 (31.0)	60 (29.4)	65 (32.7)	7.81 ± 10.20	
	4~6	107 (26.5)	63 (30.9)	44 (22.1)		
	7~12	107 (26.5)	51 (25.0)	56 (28.1)		
	≥13	64 (16.0)	30 (14.7)	34 (17.1)		
Sources of sexuality information ^‡^	Books	130 (15.4)	64 (15.5)	66 (15.4)		
	Media	306 (36.3)	162 (39.1)	144 (33.6)		
	Parents	67 (8.0)	25 (6.1)	42 (9.8)		
	Friends	221 (26.2)	115 (27.8)	106 (24.7)		
	Teachers	119 (14.1)	48 (11.6)	71 (16.6)		
Level of sexual contact *	None	64 (13.1)	35 (14.0)	29 (12.1)		
	Kissing	189 (38.6)	103 (41.2)	86 (35.8)		
	Caressing	28 (5.7)	12 (4.8)	16 (6.7)		
	Intercourse	209 (42.6)	100 (40.0)	109 (45.4)		
Sexual attitude					67.80 ± 12.65	23~115
Sexual autonomy					51.41 ± 7.63	13~65
Contraceptive self-efficacy					45.94 ± 6.64	12~60

^†^ n = 403; ^‡^ n = multiple response; * n = 490.

**Table 2:** Differences in sexual attitude, sexual autonomy, and contraceptive self-efficacy according to the participant’s characteristics (n = 499).

Characteristics	Categories	Sexual Attitude		Sexual Autonomy		Contraceptive Self-Efficacy	
		M ± SD	*t* or F(*p*)	M ± SD	*t* or F(*p*)	M ± SD	*t* or F(*p*)
Gender	Male	71.22 ± 12.84	6.34 (<0.001)	50.21 ± 8.02	−3.62 (<0.001)	44.62 ± 6.45	−4.60 (<0.001)
	Female	64.30 ± 11.48		52.65 ± 7.03		47.30 ± 6.58	
Grade	Freshman ^a^	66.19 ± 12.77	1.66 (0.175)	49.73 ± 8.02	5.38 (0.001) a < b, c **	44.23 ± 6.53	7.19 (<0.001) a < b, c, d **
	Sophomore ^b^	68.71 ± 10.86		52.70 ± 6.49		47.20 ± 6.25	
	Junior ^c^	68.92 ± 12.99		52.59 ± 7.81		46.60 ± 6.83	
	Senior ^d^	68.67 ± 14.86		51.52 ± 7.58		47.28 ± 6.51	
Family economic status	High	69.18 ± 15.36	0.63 (0.535)	51.82 ± 8.99	0.07 (0.934)	45.08 ± 6.74	0.49 (0.613)
	Middle	67.53 ± 12.13		51.36 ± 7.37		46.07 ± 6.47	
	Low	69.31 ± 14.99		51.52 ± 9.01		45.52 ± 8.17	
Parental status	Having parents	67.96 ± 12.50	0.86 (0.387)	51.38 ± 7.46	−0.30 (0.766)	45.93 ± 6.60	−0.20 (0.842)
	Parent or not having parents	66.21 ± 14.27		51.74 ± 9.41		46.14 ± 7.16	
Current residence	Parents’ home ^a^	68.79 ± 12.73	1.43 (0.240)	50.86 ± 8.07	0.73 (0.480)	45.47 ± 6.49	1.05 (0.352)
	Campus residence hall ^b^	66.05 ± 12.47		51.65 ± 7.95		45.74 ± 6.67	
	Off-campus housing ^c^	67.74 ± 12.65		51.75 ± 7.16		46.40 ± 6.75	
Date experience	No	65.54 ± 12.85	−1.96 (0.051)	50.46 ± 7.31	−1.36 (0.173)	44.42 ± 6.54	−2.52 (0.012)
	Yes	68.34 ± 12.57		51.64 ± 7.71		46.31 ± 6.62	
Number of dating experience ^†^	1 ^a^	68.85 ± 12.98	2.55 (0.055)	51.52 ± 7.91	1.62 (0.183)	45.62 ± 7.32	2.06 (0.105)
	2~3 ^b^	66.54 ± 12.00		52.18 ± 7.72		46.32 ± 6.35	
	4~5 ^c^	69.40 ± 12.75		51.97 ± 7.10		47.63 ± 6.63	
	≥6 ^d^	71.47 ± 12.78		49.57 ± 8.17		45.17 ± 6.00	
Average dating length (month) ^†^	≤3 ^a^	69.92 ± 12.16	1.25 (0.290)	50.40 ± 7.75	3.92 (0.009) a < d **	45.49 ± 6.51	2.11 (0.097)
	4~6 ^b^	68.54 ± 12.51		51.33 ± 7.13		46.02 ± 5.50	
	7~12 ^c^	67.01 ± 11.60		51.73 ± 7.43		46.54 ± 7.11	
	≥13 ^d^	67.15 ± 14.73		54.39 ± 8.43		47.98 ± 7.49	
Sources of sexuality information ^‡^	Books ^a^	64.86 ± 10.22	7.24 (<0.001) a < d, e < b, d **	51.94 ± 7.45	0.41 (0.802)	46.33 ± 6.76	1.02 (0.396)
	Media ^b^	68.41 ± 12.35		52.22 ± 7.56		46.48 ± 6.71	
	Parents ^c^	66.85 ± 12.19		51.91 ± 8.23		47.87 ± 6.82	
	Friends ^d^	69.73 ± 12.14		51.81 ± 7.80		47.14 ± 6.36	
	Teachers ^e^	63.66 ± 11.12		52.87 ± 7.76		47.21 ± 6.77	
Level of sexual contact *	None	64.46 ± 14.89	1.87 (0.132)	52.06 ± 8.60	0.26 (0.853)	45.29 ± 8.11	0.38 (0.766)
	Kissing	68.70 ± 12.45		51.05 ± 8.02		46.15 ± 6.50	
	Caressing	67.25 ± 10.98		51.50 ± 7.21		46.53 ± 6.66	
	Intercourse	68.07 ± 12.06		51.40 ± 7.08		45.76 ± 6.29	

^†^ n = 403; ^‡^ n = multiple response; * n = 490; ** Scheffé.

**Table 3:** Factors influencing contraceptive self-efficacy according to gender (n = 499).

Variables (Baseline)	Male (n = 253)									Female (n = 246)								
	Model I			Model II			Model III			Model I			Model II			Model III		
	β	t	*p*	β	t	*p*	β	t	*p*	β	t	*p*	β	t	*p*	β	t	*p*
Grade (freshman)																		
Sophomore	0.192	2.84	0.005	0.181	2.67	0.008	0.041	0.78	0.435	0.150	2.07	0.039	0.138	1.91	0.057	0.103	1.67	0.096
Junior	0.254	3.74	<0.001	0.238	3.47	0.001	0.149	2.81	0.005	0.009	0.12	0.904	0.009	0.13	0.897	−0.066	−1.07	0.286
Senior	0.094	1.43	0.152	0.092	1.40	0.160	0.060	1.19	0.233	0.175	2.51	0.013	0.159	2.29	0.023	0.122	2.06	0.040
Date experience (Yes)	0.093	1.50	0.133	0.092	1.49	0.137	0.082	1.73	0.085	0.070	1.07	0.282	0.049	0.76	0.446	0.033	0.60	0.546
Sexual attitude				0.098	1.58	0.114	0.047	0.98	0.325				0.144	2.26	0.025	0.086	1.58	0.114
Sexual autonomy							0.634	13.24	<0.001							0.517	9.55	<0.001
F(*p*)	5.33 (<0.001)			4.79 (<0.001)			36.03 (<0.001)			2.95 (0.021)			3.42 (0.005)			19.15 (<0.001)		
R^2^	0.079			0.088			0.468			0.047			0.067			0.325		
Adj R^2^	0.064			0.070			0.455			0.031			0.047			0.308		
